# Resignation of Dr. Bayless

**Published:** 1848-05

**Authors:** 


### RESIGNATION OF DOCTOR BAYLESS.

The importance which the Demonstratorship of Anatomy in the University of Louisville has assumed, will justify us in noticing the recent resignation of the gentleman who has so ably and indefatigably discharged its duties, for eight years; during which the number of students, engaged in practical anatomy, was annually, 88—115—103—127—156—187—216—256. For several years, these numbers considerably exceeded half the class of the University to which they corresponded, and during the past session, the proportion was five-eighths; which, we presume, has not been equalled in the United States, and affords high evidence of the value attached to his teachings in practical anatomy, by the students of the West and South. In common with our colleagues of the University, we regret that these onerous labors should so have affected the health of the Demonstrator as to make it expedient for him to resign. His place, however, has been filled by a promising graduate of the school, Dr. T. G. Richardson, who has acted as an assistant for two sessions.

